# Cytogenetic Profile in Monoclonal Gammopathy of Undetermined Significance, Smoldering and Symptomatic Multiple Myeloma: A Study of 1087 Patients with Highly Purified Plasma Cells

**DOI:** 10.3390/cancers15235690

**Published:** 2023-12-02

**Authors:** Guilin Tang, Yilin Wu, Pei Lin, Gokce A. Toruner, Shimin Hu, Shaoying Li, Muzaffar H. Qazilbash, Robert Z. Orlowski, Christine Ye, Jie Xu, Karen A. Nahmod, L. Jeffrey Medeiros, Zhenya Tang

**Affiliations:** 1 Department of Hematopathology, The University of Texas MD Anderson Cancer Center, 6565 MD Anderson Blvd, Houston, TX 77030, USA; peilin@mdanderson.org (P.L.); ljmedeiros@mdanderson.org (L.J.M.);; 2 Department of Biomedical Engineering, Texas A&M University, College Station, TX 77843, USA; 3 Department of Stem Cell Transplantation & Cellular Therapy, The University of Texas MD Anderson Cancer Center, Houston, TX 77030, USA; 4 Department of Lymphoma and Myeloma, The University of Texas MD Anderson Cancer Center, Houston, TX 77030, USA

**Keywords:** cytogenetic profiles, plasma cell neoplasms, refractory/relapsed myeloma, genomic complexity, heterogeneity and instability

## Abstract

**Simple Summary:**

In this study we examined the cytogenetic profile of 1087 patients with plasma cell neoplasms (PCNs) at different stages, including monoclonal gammopathy of undetermined significance (MGUS), smoldering multiple myeloma (SMM), newly diagnosed multiple myeloma (NDMM), and refractory/relapsed multiple myeloma (RRMM). Our study demonstrated that >95% of patients exhibited at least one cytogenetic abnormality detected by FISH tests and/or chromosomal analysis. The frequency of *IGH::CCND1* rearrangement was 26% in this cohort, but with no apparent differences across races, ages, or disease groups. Almost all cases with abnormal karyotypes presented a complex or composite karyotype, with most featuring five or more chromosomal abnormalities, and chromosome 1 structural abnormalities were the most prevalent (65%). The genomic complexity escalated from MGUS to SMM and further to NDMM and RRMM. Elevated frequencies of high-risk cytogenetics (59%) and the presence of subclones (48%) were particularly notable in RRMM cases.

**Abstract:**

The aim of this study was to examine the cytogenetic profiles of plasma cell neoplasms (PCNs) at various disease stages, encompassing 1087 patients with monoclonal gammopathy of undetermined significance (MGUS), smoldering multiple myeloma (SMM), newly diagnosed multiple myeloma (NDMM), and refractory/relapsed multiple myeloma (RRMM). Fluorescence in situ hybridization (FISH) analyses were conducted on highly purified plasma cell samples, revealing that 96% of patients exhibited at least one cytogenetic abnormality. The genomic complexity escalated from MGUS to SMM and further to NDMM and RRMM, largely driven by 1q gain, del(17p), *MYC*-rearrangement (*MYC*-R), del(1p), and tetraploidy. Elevated frequencies of high-risk cytogenetics (59%), 1q gain (44%), and del(17p) (23%), as well as the presence of subclones (48%), were particularly notable in RRMM cases. *IGH::CCND1* was observed in 26% of the cases, with no apparent variations across races, ages, or disease groups. Concurrent chromosomal analysis with FISH revealed that the incidence of abnormal karyotypes was strongly correlated with the extent of neoplastic plasma cell infiltration, genomic complexity, and the presence of specific abnormalities like del(17p) and *MYC*-R. Approximately 98% of the cases with abnormal karyotypes were complex, with most featuring five or more abnormalities. Chromosome 1 structural abnormalities were the most prevalent, found in 65% of cases. The frequent presence of subclones and composite karyotypes underscored the genomic heterogeneity and instability in this cohort.

## 1. Introduction

Almost all patients with multiple myeloma (MM) evolve from a premalignant stage termed monoclonal gammopathy of undetermined significance (MGUS) [1,2]. MGUS progresses to MM at a rate of 1% per year [3]. In some patients, an intermediate and asymptomatic but more advanced stage termed smoldering multiple myeloma (SMM) can be recognized [4]. SMM progresses to MM at a rate of approximately 10% per year over the first 5 years following diagnosis, 3% per year over the next 5 years, and 1.5% per year thereafter [4]. The risk of progression from MGUS or SMM to MM is greatly influenced by the underlying cytogenetic abnormalities: patients with t(4;14), del(17p), and 1q gain (1q+) are at a higher risk of progression from MGUS or SMM to MM [5,6,7].

Multiple factors contribute to patient prognosis and survival, including host characteristics, tumor burden (stage), cytogenetic abnormalities, and response to therapy. Among several risk stratification systems, the International Staging System (ISS) [8] and the revised ISS (R-ISS) [9] plus the Mayo Clinic mSMART risk stratification [10] (www.msmart.org (lastly accessed on 30 August 2023)) are the most widely applied in clinical practice. The ISS, developed in 2005, ref. [8], is based on serum β2-microblobulin and albumin levels. The R-ISS, developed in 2015, ref. [9], is derived from the ISS but incorporates three high-risk cytogenetic abnormalities, t(4;14), t(14;16), and del(17p), as well as the elevated serum level of lactate dehydrogenase (LDH). During the past couple of years, more and more data have shown that 1q+ is an independent poor prognostic factor and a high-risk factor for progression from SMM to MM [11,12,13,14,15]. As a result, with the recent Second Revision of the ISS (R2-ISS) [16], 1q+ has been added to the risk score system. In the Mayo Additive Staging System (MASS), the high-risk *IGH* translocations t(4;14), t(14;16), and t(14;20); 1q+; del(17p); ISS stage III; and elevated serum LDH are identified as independent high-risk factors associated with decreased overall survival [17].

More than 90% of plasma cell neoplasms (PCNs) harbor at least one chromosomal abnormality detected via fluorescence in situ hybridization (FISH) and/or conventional chromosomal analysis (karyotyping). The cytogenetic abnormalities feature trisomies of odd-numbered chromosomes (with +9, +11, +15 being most common) and/or *IGH* rearrangement (*IGH*-R) [18,19]. Trisomies, del(13q), and *IGH*-R in t(4;14), t(11;14), and t(14;16) are considered to be primary cytogenetic abnormalities and occur at the time of the establishment of MGUS. Other cytogenetic changes, termed secondary cytogenetic abnormalities, arise along the disease course and/or during progression, including 1q+, del(1p), del(17p), and *MYC* rearrangement (*MYC*-R) [18,19]. Both primary and secondary cytogenetic abnormalities can affect the disease course, patient response to therapy, and outcomes. However, conducting large-scale cytogenetic profiling studies of PCNs poses substantial technical challenges due to the difficulty of obtaining high-purity plasma cells and the generally low proliferation rate of plasma cells under in vitro culture conditions.

In this study, we analyzed the cytogenetic profiles of 1087 patients with MGUS, SMM, newly diagnosed MM (NDMM), and refractory/relapsed MM (RRMM) via karyotyping and FISH studies. About 91% of the specimens underwent the enrichment of plasma cells (EPC) prior to FISH analysis, while the remaining 9% of cases all had PC >20% in the bone marrow (BM).

## 2. Materials and Methods

### 2.1. Patients

Patients with MGUS, SMM, and MM who were diagnosed and managed at our institution from 2 January 2021 through 31 May 2023 formed the study cohort. The diagnosis of MGUS, SMM, and MM were based on the World Health Organization Classification of Tumors of Hematopoietic and Lymphoid Tissues [20]. Clinical information was retrieved from the electronic medical records. This study was approved by the Institutional Review Board of MD Anderson Cancer Center and was conducted in accordance with the Declaration of Helsinki.

### 2.2. Assessment of Plasma Cell Involvement

The degree of plasma cells’ (PCs) involvement was evaluated via: (1) differential count (diff) on BM aspirate smears; (2) immunohistochemistry for CD138 on BM core biopsy and/or clot sections; and (3) flow cytometric immunophenotypic analysis that included markers specific to CD19, CD27, CD38, CD45, CD56, CD81, CD117, CD138, cytoplasmic (cyto) kappa, and lambda immunoglobin light chain as described previously [21]. Aberrant PCs were distinguished from normal PCs based on their aberrant expression of two or more of the following antigens: CD19−, CD27−/low, CD38+/low, CD45−, CD56+, CD81−/low, CD117+, and/or monotypic light chain expression. The percentages (median) of aberrant (neoplastic or clonal) PCs out of total PCs were 85% (ranges, 25% to 100%), 95% (ranges, 30% to 100%), 97% (ranges, 59% to 100%), and 96% (ranges, 25% to 100%) for MGUS, SMM, NDMM, and RRMM, respectively.

### 2.3. Plasma Cell Purification

Enrichment of plasma cells (EPC) was conducted on BM aspirates when PCs were ≤20%. Meanwhile, the aberrant (neoplastic) PCs comprised at least 0.05% of the total analyzed cells, and these aberrant PCs made up 25% or more of the total plasma cell population (estimated via flow cytometry). Enrichment was achieved by using RoboSep (Stemcell Technologies, Vancouver, BC, Canada) and anti-CD138 magnetic microbeads according to the manufacturer’s instructions. Briefly, 2 mL BM aspirate was mixed with 8 mL D-PBS then filtered through 70 um strainer. The filtered specimen was spun down at 300× *g* for 10 min and the supernatant was carefully removed. The cell pellet was then resuspended in 2 mL D-PBS. After adding 2 mL red blood cell lysis buffer to the suspension, the specimen tube was placed in the RoboSep machine and the procedure was run accordingly. The positive selected cells (PCs) were collected in 1 mL D-PBS. To estimate the purity of plasma cells in the post-enrichment samples, we prepared cytospin slide by using 100 uL post-enrichment collection. The cytospin slide then underwent Wright-Giemsa stain, and the purity of plasma cells was estimated via morphological examination (plasma cells out of total cells). Overall, the purity of plasma cells in post-enrichment specimens ranged from 60% to nearly 100%. The left-over (~900 uL) post-enrichment solution was spun down (at 300× *g*, 8 min), and the cell pellet was then resuspended in fixation buffer (3:1 methanol: acetic acid) for FISH analysis. Based on our laboratory algorithm, if BM aspirate is insufficient for both karyotyping and EPC/FISH, the latter takes priority unless a diagnosis of a myelodysplastic syndrome is suspected.

### 2.4. FISH Analysis

FISH analysis was either performed on cultured cells when PCs were >20% or on EPC following manufacturer’s instructions (Abbott Molecular, Abbott Park, IL, USA). Eight dual-color FISH probe sets were included in our myeloma FISH panel as recommended by the International Myeloma Working Group (IMWG) [22]: four probe sets for copy number changes, including 1p32.3 (*CDKN2C*)/1q21 (*CKS1B*), 9p21 (*CDKN2A*)/CEP9, 13q14 (*RB1*)/13q34, and 17p13 (*TP53*)/CEP17; and four for gene rearrangements (which also reflect gene copy number alterations), including t(4;14)(p16;q32)/*IGH::FGFR3* (dual-fusion); t(11;14)(q13;q32)/*IGH::CCND1* (dual-fusion); t(14;16)(q32;q23)/*IGH::MAF* (dual-fusion); and 8q24/*MYC* (break-apart). *IGH* (break-apart) FISH testing was reflexed when *IGH* exhibited three signals but was not fused with *CCND1, FGFR3,* or *MAF*. At least 100 cells were counted for each FISH probe set. The cut-off values for common abnormal patterns established in our Clinical Cytogenetics Laboratory are summarized in Supplemental S1. A 1q gain was defined as 3~4 copies of *CKS1B*, and 1q amplification (amp) was defined as 5 or more copies of *CKS1B* (lumped as 1q+ if not otherwise specified in this study). The presence of subclone(s) was defined by the following criteria: (1) co-existence of two or more abnormalities in the same gene/locus, e.g., different copies of *CKS1B* are detected in one specimen; (2) simultaneous presence of both diploid and aneuploid populations; and (3) the difference in clonal size exceeds 30%.

### 2.5. Chromosomal Analysis

Chromosomal analysis was performed on metaphase cells prepared from BM aspirates cultured for 72 h with lipopolysaccharide (LPS) and/or 24 h without mitogens using standard techniques. Twenty Giemsa-banded metaphases were analyzed, and the results were reported using the International System for Human Cytogenetic Nomenclature (ISCN 2020). Cases with 3 or more unrelated chromosomal abnormalities (at least one structural abnormality) were considered to have a complex karyotype. Structural abnormalities included deletion (del), duplication (dup), additional material of unknown origin (add), translocation (t), isochromosome, etc. The karyotype was arbitrarily divided into hypodiploidy (chromosomal number < 46); diploidy (chromosomal number = 46); hyperdiploidy (chromosomal number > 46); and aneuploidy (mainly tetraploidy) in this study.

## 3. Results

### 3.1. Patients

This study cohort included 1087 patients: 137 with MGUS, 205 with SMM, 252 with NDMM, and 493 with RRMM. The demographic information (race, age, and gender) is summarized in Table 1. Of these patients, 56% were male and 44% were female; and 68% were Caucasian, 21% were African American, 3% were Asian, and 7% were other races (American Indian, Alaska Native, Native Hawaiian, or other Pacific Island, and others not specified). The age distributions in <50, 50–59, 60–69, 70–79, and >80 years patients were 7%, 20%, 36%, 29%, and 9%, respectively.

All patient specimens were assessed using a MM FISH panel: 986 (91%) on EPC and 101 (9%) on cultured cells. A total of 802 patients had concurrent karyotyping, while 285 other cases had insufficient BM specimen and it was not possible to do karyotyping. The percentage of plasma cells in bone marrow aspirate (by differential count), the corresponding FISH analysis and karyotyping, and the cases with abnormal results are summarized in Table 2.

### 3.2. Cytogenetic Abnormalities Detected via FISH Analysis

**Figure 1:**
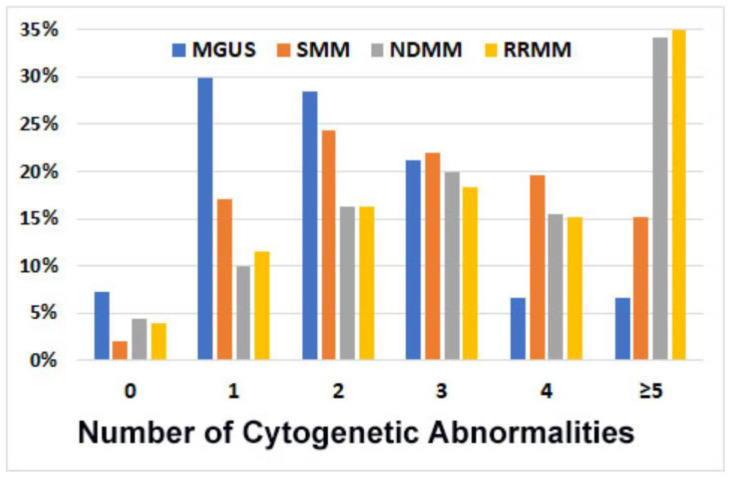
Number of cytogenetic abnormalities (per case) detected via FISH analysis in 1087 patients. In total, 93% of cases with monoclonal gammopathy of undetermined significance (MGUS), 98% of smoldering myeloma (SMM), 96% of newly diagnosed myeloma (NDMM), and 96% of refractory/relapsed myeloma (RRMM) showed cytogenetics abnormalities; the number of abnormalities was markedly higher for NDMM and RRMM.

Number of cytogenetic abnormalities. Overall, 1043 patients (96%) showed at least one PCN-related cytogenetic abnormality: 93% in MGUS patients, 98% in SMM, and 96% in NDMM and RRMM. Most patients in the MGUS group (65%) showed two or fewer cytogenetic abnormalities, whereas most of the MM patients (68%) showed three or more abnormalities. Furthermore, 34~35% of patients with MM showed five or more abnormalities, versus only 15% in SMM and 7% in MGUS patients. The average number of cytogenetic abnormalities per case was 1.8 for MGUS, 3 for SMM, and 4.2 for MM. The cytogenetic complexity was similar between RRMM and NDMM cases (Figure 1).

**Figure 2:**
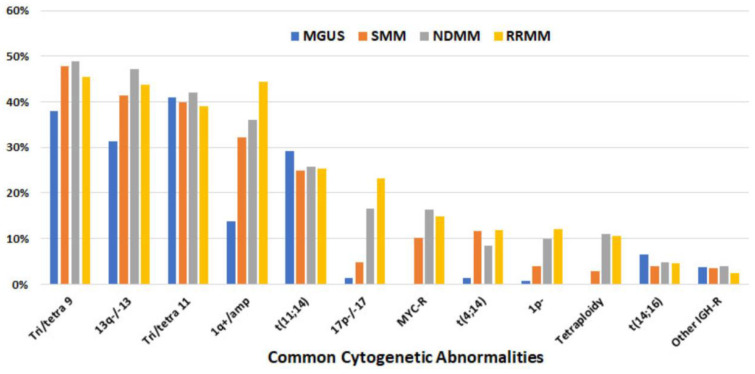
Frequency of common cytogenetic abnormalities detected via FISH (listed in descending order based on overall frequency) in 1087 patients.

Common cytogenetic abnormalities. Overall, the most common abnormalities detected via FISH were trisomy 9/tetrasomy 9 (tri/tetra9), del(13q)/−13, and tri/tetra11, present in ≥40% of patients and at a similar frequency among MGUS, SMM, NDMM, and RRMM. The other two cytogenetic abnormalities present at a similar frequency with MGUS, SMM, and MM were t(11;14) and t(14;16), present in ~26% and ~5% of patients, respectively. On the other hand, the frequency of 1q+/amp increased from 14% for MGUS to 44% for RRMM. A similar pattern was also observed in del(17p)/−17 (from 1% for MGUS to 23% for RRMM) and del(1p) (from 1% in MGUS to 12% in RRMM). Other cytogenetic abnormalities, including t(4;14), *MYC*-R, and tetraploidy, were rare or absent with MGUS but detected in 8% to 12% of RRMM cases (Figure 2, Table 3).

**Figure 3:**
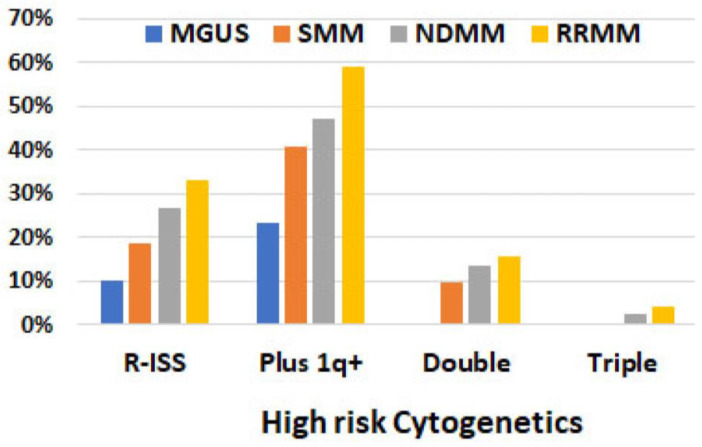
Frequency of high-risk cytogenetics detected via FISH analysis in 1087 patients. R-ISS included t(4;14), t(14;16), and del(17p). “Plus 1q+” refers to including 1q+ on the top of three high-risk factors in R-ISS. Double: two co-existing risk factors (including 1q+). Triple: three co-existing risk factors (including 1q+).

High-risk cytogenetic abnormalities. Based on R-ISS, high-risk cytogenetics, which included t(4;14), t(14;16), and del(17p), were detected in 10% of MGUS, 19% of SMM, 27% of NDMM, and 33% of RRMM patients. The most common high-risk cytogenetic abnormalities were t(14;16) in MGUS, t(4;14) in SMM, and del(17p) in NDMM and RRMM patients (Table 3, Figure 3). When 1q+/amp was included in the high-risk group, the frequency of high-risk cytogenetic abnormalities increased to 23%, 41%, 47%, and 59% for MGUS, SMM, NDMM, and RRMM, respectively. Double high-risk was not detected in MGUS patients, but was detected in 10%, 13%, and 16% of SMM, NDMM, and RRMM ones, respectively. Triple high-risk was not detected in either MGUS or SMM patients but was detected in 2% of NDMM and 4% of RRMM ones. (Figure 3).

Subclones. Subclones were detected in 41% of the cases in this cohort, with 14% in MGUS, 36% in SMM, 48% in NDMM, and 48% in RRMM patients. The cytogenetic abnormalities detected as subclones in MGUS patients were limited to 1q+, tetrasomy 9, −13, and loss of *IGH.* However, the abnormalities in subclones were highly variable in SMM, NDMM, and RRMM patients, including 1q+/amp (most common), aneusomies of chr9, chr13, and chr17, del(17p), *MYC*-R, losses of *CDKN2C*, *IGH*, *FGFR3*, *MAF*, and aneuploidy. Sixty cases with RRMM (12%) showed a spectrum of 1q copies (up to 12 copies) within one specimen.

Cytogenetic abnormalities among demographic groups (Table 1). There was no apparent difference between Caucasian and African American in aspects of disease stages, frequencies of t(4;14); t(11;14), t(14;16), and del(17p), and high-risk cytogenetics. Other races were not compared due to the limited number of patients included in this cohort.

Cases with one or two cytogenetic abnormalities. A single cytogenetic abnormality was detected in 158 patients (15%). The most common single abnormality was t(11;14) (*n* = 113, 10.4%), followed by trisomy 11 (*n* = 15), trisomy 9 (*n* = 10), and del(13q) (*n* = 6). Two abnormalities were detected in 210 patients (19%), with the most common being trisomy 11 plus trisomy 9 (*n* = 45), t(11;14) plus del(13q) (*n* = 43), t(11;14) plus 1q+ (*n* = 16), and t(4;14) plus del(13q) (*n* = 13).

### 3.3. Cytogenetic Abnormalities Detected via Karyotyping

Frequency of abnormal karyotype. Karyotyping was performed in 802 patients. Of these, 623 patients showed a normal karyotype (including 18 cases with minus Y only); 164 (21%) showed a PCN-related abnormal karyotype, of which 161 (98.2%) had a complex karyotype, 2 had hyperdiploidy (only chromosome gains), and 1 had 2 abnormalities (Supplemental S2). The yield of an abnormal karyotype was highly associated with the degree of BM involvement by PCN. When PCs constituted ≤5% of the total cells, only 4% of the patients showed an abnormal karyotype, although a striking 94% of them showed at least one abnormality detected via FISH. As the percentage of PCs in BM increased, so did the frequency of abnormal karyotypes: 9% in cases with 6~10% of PCs; 16% in cases with 11~20% of PCs; and 63% in cases with PCs >20% (Table 2). The yield of PCN-related abnormal karyotypes was also associated with the disease classification: 0% of MGUS, 11% of SMM, and 27% of MM patients (Table 4). Of note, 15 patients showed an abnormal karyotype unrelated to a PCN and attributable to co-existing myelodysplastic syndrome (MDS), chronic myeloid leukemia (CML), chronic lymphocytic leukemia (CLL), and small clones of unknown clinical significance, which were exemplified by −5/del(5q), −7, del(20q) in MDS, t(9;22) in CML, and trisomy 12 in CLL.

**Figure 4:**
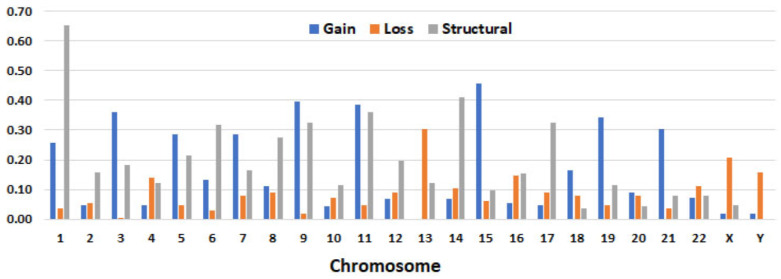
Aneusomies (gain, loss) and/or structural abnormalities detected via chromosomal analysis in 163 patients. Detailed information listed in Supplemental S2. (1-22, X, Y are chromosomes).

Common chromosomal abnormalities. Based on the chromosomal number in the main clone, the karyotypes could be largely divided into four groups: hypodiploid (27%), diploid (8%), hyperdiploid (61%), and aneuploid (4%). Most of the cases showed five or more abnormalities. The most involved chromosomes were chr1 (67%), chr11 (67%), chr9 (66%), chr15 (58%), chr5 (52%), and chr14 (50%). Structural abnormalities were most frequently detected in chr1 (65%), followed by chr14 (41%) and chr11. Among trisomies, +15 was the most common (46%), followed by +9, +11, and other odd number chromosomes, except for chr17 and chr13 (no cases showed +13). On the other hand, monosomy 13 was the most common aneusomy resulting from loss (30%) (Table 5 and Figure 4). Marker chromosomes (mar) were detected in 114 patients (65%) and a composite karyotype (cp) was detected in 109 patients (62%). The detailed chromosomal abnormalities detected via karyotyping in each case are summarized in Supplemental S2.

Correlation of FISH results with karyotype. The yield of an abnormal karyotype was closely associated with the number of cytogenetic abnormalities detected via FISH; the greater number of FISH abnormalities, the higher thr yield of an abnormal karyotype (Table 4). Cases harboring del(17p) and *MYC*-R (detected via FISH) were more likely to have an abnormal karyotype: 44% of cases with del(17p) and 43% of cases with *MYC*-R showed an abnormal karyotype. In contrast, cases with t(11;14) (detected via FISH) were the least likely to have an abnormal karyotype, occurring only in 13% of cases (Table 4). Due to the limited coverage of the genome by FISH probes, karyotyping picked up more cytogenetic abnormalities in the cases exhibiting an abnormal karyotype. These phenomena were more evident in 86 cases that only showed chromosomal gains via FISH. Among them, 76 had a normal karyotype, 2 were hyperdiploid (without structural abnormalities), and 8 patients had a hyperdiploid karyotype plus structural abnormalities, which was more consistent with a complex karyotype.

## 4. Discussion

Interphase FISH and chromosomal analysis have been widely used to detect cytogenetic abnormalities in plasma cell neoplasms. However, given the typically low proliferation rate of plasma cells under in vitro culture conditions, generating metaphase cells for karyotyping is challenging in most MM cases, and even more so in SMM or MGUS cases. Additionally, FISH can yield “false negative” results when BM involvement by neoplastic PCs is low. To enhance FISH detection sensitivity, the American College of Medical Genetics recommends enriching the PCs using magnetic beads and/or flow cytometry sorting before FISH analysis [23]. This enrichment is crucial for the specimens with very low PCN involvement and for detecting small subclones. In this cohort, EPC was performed in approximately 90% of the cases, enabling us to identify PCN-related abnormalities in 96% of the cases, even in specimens with very low-level involvement (as low as 0.02% aberrant PCs by flow), and detect subclones in about 40% of the patients.

The similar frequency of t(11;14), t(14;16), +9, +11, and del(13q) detected in patients with MGUS, SMM, and MM supports the theory that these cytogenetic abnormalities are primary events, occurring during an early stage of PCN, and are unlikely to play roles in disease progression. We observed a slightly increased frequency of tetrasomy 9 and −13 with SMM and MM, which was likely associated with clonal evolution. Conversely, t(4;14) was more commonly detected with SMM and MM, indicating that t(4;14) as a primary event poses a higher risk of progression to SMM and MM [20,24]. We detected a slightly higher frequency (26%) of t(11;14) in comparison with other studies [25]; this difference might be attributable to FISH being performed on highly purified PCs, with around 10% of cases showing t(11;14) as the sole abnormality. In addition, we did not observe a disparity of t(11;14) among the race, gender, or age groups. None of the patients in this cohort exhibited co-existing t(11;14), t(4;14), or t(14;16), suggesting that these three “primary” events are mutually exclusive.

The frequency of 1q+ and the copy number of 1q increased steadily from MGUS to SMM, NDMM, and RRMM. The frequency of 1q+ detected in MM in our cohort was similar to what has been reported by other studies [6,26]. For MGUS, cases with 1q+ only showed three copies of *CKS1B*; in RRMM, ~40% of the cases with 1q+ showed four or more *CKS1B* copies, including 15% of the cases that showed *CKS1B* amplification (five or more copies), and 12% of the cases that showed subclones with different *CKS1B* copies, which is consistent with clonal evolution and disease progression [27,28,29]. Patients with 1q+ were noted to have frequent and common breakpoints in the pericentromeric heterochromatin region [27,28], causing the repetitive “jumping” translocation of the whole long arm to other chromosomes and duplication [28]. Frequent copying of *CKS1B* leads to CKS1B overexpression [30], which has been shown to promote MM cell growth by activating cyclin-dependent kinases and reducing the level of the tumor suppressor p27Kip1 [13]. Subsequently, CKS1B was found to promote MM cell drug resistance via the upregulation of the *STAT3* and *MEK/ERK* pathways [14]. Despite 1q+ having been considered to be an independent poor prognostic factor for MM patient progression-free survival and overall survival [6,11,12,13,15,16], 1q+ has not been uniformly adopted as a high-risk cytogenetic abnormality in the guidelines [9].

Chromosome 17p deletion is another cytogenetic abnormality that was more common in RRDD, from 1% in MUGS to 23% in RRMM. All cases with del(17p) showed three or more abnormalities detected via FISH, supporting the association of del(17p)/*TP53* deletion with a complex karyotype and genomic instability. We also observed a high frequency of abnormal karyotypes in the presence of del(17p), suggesting that del(17p) may confer proliferative advantages to neoplastic PCs. Del(17p) is consistently recognized as a high-risk factor across all guidelines [18].

*MYC*-R was not detected in any case with MGUS but was present in 10% of SMM and 15% of MM cases. Other studies have shown a frequency of *MYC*-R in MM varying from 8% to 50%, depending on the methods used for detection [31,32,33,34]. Only about 25% to 38% of MM cases with *MYC*-R had immunoglobulin gene partners [31,32], which is much less frequent than with Burkitt lymphoma (near 100%) and high-grade B-cell lymphoma (75–80%) [31]. The recurrent non-IG rearrangement partners for *MYC* detected in MM included: 1p12 (*FAM46C*), 6p24.3 (*TXNDC5*), 6q21 (*FOXO3, PRDM1*), 8q24.13/*NSMCE2*, 11q13.3 (*CCND1*), and 7p21.3 (*GLCCI1*). Most of these partner genes are also super-enhancers [33]. In our cohort, *MYC* partners included *IGH* (most common, *n* = 8), *IGL*, *IGK*, *NSMCE2*, *TXNDC5*, and *CCND1*, but the partner was uncertain for most cases. The breakpoints showed a much wider range around the *MYC* locus during *MYC*-R, and the breakpoint clusters were not as distinct in MM as the clusters in lymphomas [31], which may explain the low detection rate of *MYC*-R in MM via FISH [31]. *MYC*-R is associated with elevated serum levels of beta-2-microglobin and a high disease burden and is an independent adverse prognostic factor in patients with NDMM [32,34].

Aneuploid (mainly near-tetraploidy in our cohort) clones were not detected in MGUS, were rare (~3%) in SMM, and were found in about 10% of MM cases in this cohort. Tetraploidy was reported in 6% to 10% of MM patients in other studies [35,36]. Most cases with aneuploid also had near-diploidy and had aneuploidy in sub-clones: ~80% via FISH analysis and 61% via chromosomal analysis, which also support the presence of clonal evolution in these cases. Aneuploidy was associated with pleomorphic/anaplastic plasma cells, a high-grade MM morphology. Other studies have reported that tetraploidy is associated with resistance to lenalidomide and bortezomib treatment, disease relapse, and a poor prognosis in MM patients [36,37,38].

The poor growth of PCs under in vitro culture conditions leads to difficulty in obtaining metaphase cells for karyotyping, especially when the BM involvement by PCs is low. This fact likely explains why the karyotypes of MGUS and SMM have been rarely reported. In our cohort, no case with MGUS and only 11% of cases with SMM showed an abnormal karyotype, with chromosomal gains as the major abnormalities. The likelihood of producing metaphase cells from PCN was highly associated with high-level BM involvement, the presence of del(17p) and *MYC*-R, and the complexity of the genome. Almost all cases with an abnormal karyotype showed a complex karyotype, and most showed abnormalities on chr1 (structural abnormalities in 65% of cases), chr11 and chr9 (both trisomy and structural abnormalities), and +15. A marker chromosome and composite karyotype were detected in more than 60% of the patients, corresponding to the high frequency of subclones detected via FISH. These findings highly suggest clonal heterogeneity and genome instability in PCNs showing an abnormal karyotype.

## 5. Conclusions

In summary, our FISH panel identified cytogenetic abnormalities in 96% of PCNs. We observed low genomic complexity in MGUS patients, with an increasing degree of complexity for SMM and MM, and the later-acquired del(17p), *MYC*-R, and high-copy number of 1q which enable proliferative advantages in neoplastic plasma cells. In the cases where a PCN-related karyotype was produced, the karyotype was often highly complex, with structural abnormalities of chr1 being the most common. To enhance our understanding of the whole genomic profile of PCNs, future studies could incorporate a DNA-based assay that doesn’t require metaphase cells but covers the entire genome, such as optical genome mapping.

## Figures and Tables

**Table 1:** Demographic features, diagnosis, common *IGH* rearrangements, del(17p), and high-risk cytogenetics (based on R-ISS).

	Case No	Total (%)	MGUS	SMM	NDMM	RRMM	t(11;14)	t(4;14)	t(14;16)	del(17p)	HR- R-ISS	HR-Plus 1q+
**Races**												
Caucasian	744	68%	14%	19%	24%	43%	27%	10%	5%	16%	27%	48%
African American	225	21%	11%	18%	22%	49%	23%	7%	5%	13%	23%	51%
Asian	38	3%	16%	21%	21%	42%	26%	13%	0%	18%	26%	42%
Others	80	7%	6%	19%	18%	58%	24%	15%	5%	13%	26%	49%
**Gender**												
Male	613	56%	11%	18%	23%	48%	27%	9%	4%	15%	24%	46%
Female	474	44%	14%	20%	23%	42%	25%	11%	6%	17%	28%	52%
**Age (years)**												
<50	72	7%	8%	21%	25%	46%	28%	13%	1%	18%	26%	47%
50–59	213	20%	14%	21%	22%	44%	23%	13%	5%	15%	28%	50%
60–69	391	36%	13%	16%	21%	49%	25%	10%	7%	16%	28%	50%
70–79	311	29%	13%	20%	25%	43%	28%	8%	4%	14%	24%	47%
>80	100	9%	11%	22%	26%	41%	28%	6%	2%	17%	22%	46%

**Table 2:** Plasma cell counts in the pre-enrichment bone marrow; FISH analysis (on EPC or on cultured cell) and the cases showing abnormal results; karyotyping and the cases showing abnormal karyotype.

PC (%)	FISH					Chromosomal Analysis				
	Case No	On EPC	On Culture	Abnormal (Case No)	Abnormal %	Case No	Normal Karyotype *	Abnormal Karyotype	Abnormal %	PCN-UR
0	53	53	0	46	87%	34	32	0	0%	2
1	127	127	0	114	90%	96	89	4	4%	3
2	106	106	0	103	97%	77	72	2	3%	3
3~5	208	208	0	197	95%	157	148	7	4%	2
6~10	183	183	0	180	98%	130	114	12	9%	4
11~20	150	150	0	148	99%	116	96	19	16%	1
>20	260	159	101	255	98%	192	72	120	63%	0
Total	1087	986	101	1043	96%	802	623	164	20%	15

* Includes the cases with minus Y as the sole abnormality. EPC: enrichment of plasma cells; PC: plasma cells; PCN-UR: plasma cell neoplasm-unrelated abnormal karyotype.

**Table 3:** Frequency of common cytogenetic abnormalities in 1087 patients with MGUS, SMM, NDMM, and RRMM, evaluated via FISH analysis.

	MGUS	SMM	NDMM	RRMM	Total
Tri/tetra 9	38%	48%	49%	45%	46%
Del(13q)/−13	31%	41%	47%	44%	43%
Tri/tetra 11	41%	40%	42%	39%	40%
1q+/amp	14%	32%	36%	44%	36%
t(11;14)	29%	25%	26%	25%	26%
del(17p)/−17	1%	5%	17%	23%	15%
*MYC*-R	0%	10%	16%	15%	12%
t(4;14)	1%	12%	8%	12%	10%
1p−	1%	4%	10%	12%	9%
Tetraploidy	0%	3%	11%	11%	8%
t(14;16)	7%	4%	5%	5%	5%
Other *IGH*-R	4%	3%	4%	2%	3%

**Table 4:** The likelihood of yielding an abnormal karyotype (among 802 patients) is associated with disease stages and cytogenetic abnormalities detected via FISH.

	Karyotype			
	Total	Normal	Abnormal *	Frequency of Abnormal Karyotype
**Diagnosis**				
MGUS	106	103	0	0%
SMM	152	132	16	11%
NDMM	193	139	51	26%
RRMM	351	249	97	28%
**Number of abnormalities detected via FISH**				
None	36	36	0	0%
1	120	110	5	4%
2	158	143	13	8%
3	167	135	27	16%
4	132	87	44	33%
5 or more	189	112	75	40%
**Abnormalities (detected via FISH)**				
del(17p)/−17	117	63	52	44%
*MYC*-R	93	53	40	43%
1q+	300	203	96	32%
t(4;14)	75	52	23	31%
t(14;16)	32	24	8	25%
trisomy 9	350	258	87	25%
del(13q)/−13	331	257	70	21%
t(11;14)	217	183	28	13%

* Only plasma cell neoplasm-related abnormal karyotype.

**Table 5:** The frequency of cytogenetic abnormalities detected via chromosomal analysis in 164 patients in a descending order.

Total		Gain-Aneusomy		Loss-Aneusomy		Structural Abnormality	
Chrom No	Abnormal	Chrom No	Abnormal	Chrom No	Abnormal	Chrom No	Abnormal
1	67%	15	46%	13	30%	1	65%
11	67%	9	40%	X	21%	14	41%
9	65%	11	38%	Y	16%	11	36%
15	58%	3	36%	16	15%	9	32%
5	52%	19	34%	4	14%	17	32%
14	50%	21	30%	22	11%	6	32%
3	50%	5	29%	14	10%	8	27%
19	46%	7	29%	8	9%	5	21%
7	45%	1	26%	12	9%	12	20%
6	44%	18	16%	17	9%	3	18%
17	42%	6	13%	7	8%	7	16%
13	42%	8	11%	18	8%	2	16%
8	41%	20	9%	20	8%	16	15%
21	39%	22	7%	10	7%	4	12%
16	32%	12	7%	15	6%	13	12%
12	31%	14	7%	2	5%	10	12%
4	29%	16	5%	5	5%	19	12%
18	27%	2	5%	11	5%	15	10%
22	27%	4	5%	19	5%	21	8%
X	26%	17	5%	1	4%	22	8%
2	23%	10	4%	21	4%	X	5%
10	22%	X	2%	6	3%	20	4%
20	21%	Y	2%	9	2%	18	4%
Y	18%	13	0%	3	1%	Y	0%

## Data Availability

The datasets used and/or analyzed during the current study are available from the corresponding authors upon reasonable request.

## Supplementary Materials

The following supporting information can be downloaded at: https://www.mdpi.com/article/10.3390/cancers15235690/s1, Supplemental S1: Major Abnormal FISH Signal Patterns with Corresponding Abnormalities and Cut-off Values; Supplemental S2: Detailed cytogenetic abnormalities detected in 163 patients who showed abnormal karyotype by chromosomal analysis.
