# Supplementary material for: Cytogenetic Profile in Monoclonal Gammopathy of Undetermined Significance, Smoldering and Symptomatic Multiple Myeloma: A Study of 1087 Patients with Highly Purified Plasma Cells
Source: Cancers (Basel). 2023 Dec 2;15(23):5690. doi: 10.3390/cancers15235690 (PMC10705751; doi:10.3390/cancers15235690)
Supplement: Supplementary file 1 [file cancers-15-05690-s001.zip › Supplemental S1.pdf]

Supplemental S1: Major Abnormal FISH Signal Patterns with Corresponding Abnormalities and Cut-off Values.

**FISH probes/ Signal patterns**	**Abnormality**	**Cut-off (%)**
CDKN2C/CKS1B		
2R1G	Loss of one copy of CDKN2C	<6.5
3R2G	Gain of one copy of CKS1B	<9.9
4R2G	Gain of two copies of CKS1B	<3.5
3R1G	Gain of one copy of CKS1B and loss of one copy of CDKN2C	<1.4
IGH/FGFR3		
1R1G2F	IGH::FGFR3 rearrangement	<0.5
3R2G	Gain of one copy of FGFR3	<1.0
2R3G	One extra signal of IGH or IGH rearrangement	<3.0
MYC		
1R1G1F	MYC rearrangement	<4.7
1R2F	Gain of 5'MYC / MYC rearrangement	<0.7
3F	Gain of one copy of MYC	<4.0
CDKN2A/CEP9		
3R3G	Trisomy 9	<0.5
4R4G	Tetrasomy 9	<2.2
1R2G	Loss of one copy of CDKN2A	<3.8
IGH/MYEOV-CCND1		
1R1G2F	IGH::CCND1/MYEOV rearrangement	<0.5
3R2G	Gain of one copy of MYEOV-CCND1	<3.8
2R3G	One extra signal of IGH or IGH rearrangement	<10.6
RB1/13q34		
1R2G	Loss of one copy of RB1	<5.1
1R1G	Monosomy 13	<5.1
IGH/MAF		
1R1G12F	IGH::MAF rearrangement	0.0
2R3G	One extra signal of IGH or IGH rearrangement	<1.4
1R2G	Loss of one copy of MAF	<0.5
TP53/CEP17		
1R2G	Loss of one copy of TP53	<7.9
1R1G	Monosomy 17	<4.9
